# Higher Star, Better Performance in US Nursing Homes during the Covid-19 Pandemic ?

**DOI:** 10.1093/geroni/igab046.2717

**Published:** 2021-12-17

**Authors:** Lei Yu, Xiao Qiu, Tara Rose

**Affiliations:** 1 Miami University, Oxford, Ohio, United States; 2 University of Southern California, Los Angeles, California, United States

## Abstract

The Covid-19 pandemic has brought terrible difficulties to nursing homes, as they were locations with the highest number of confirmed Covid-19 cases and deaths in the US. The Centers for Medicare & Medicaid Services (CMS) applies the Five-Star Quality Ratings to indicate the quality of care in nursing homes based on health inspection survey, staffing, and resident outcome. Studies to date have contradictory findings regarding the relationship between nursing home reported Quality Ratings and Covid-19 cases and deaths based on US regional data. The purpose of this study is to examine whether nursing homes’ Quality Ratings were related to the total number of resident Covid-19 cases and deaths at the US National level. The study examined US nursing homes (N=13,494) linked with CMS Nursing Home Compare data and Covid-19 Nursing Home data. Using multiple linear regression analyses, results showed nursing home Quality Ratings were significantly associated with Covid-19 residents’ cases and deaths controlling for ownership type, size, occupation rate, and years of operation (p<.001; p<.001). Five-star nursing homes were less likely to have Covid-19 cases and deaths. Further, comparing lower Star Ratings nursing homes, 1-Star nursing homes showed no significant difference to 2-Star and 3-Star nursing homes when examining Covid-19 cases and deaths. Overall, the Five-Star Quality Ratings is a useful measure when investigating nursing homes’ performance during the Covid-19 pandemic. Future policymakers and administrators should also focus on nursing homes with lower star ratings when improving the quality of nursing homes, particularly with regard to resident health.

